# Multi-Scale Feature Fusion Based RT-DETR for Tomato Leaf Disease Detection in Complex Backgrounds

**DOI:** 10.3390/s25237275

**Published:** 2025-11-28

**Authors:** Shaohuang Bian, Shan Su, Jun Zhou, Chengxi Yi, Feng Huang

**Affiliations:** 1 College of Information and Electronic Engineering, China Agricultural University, Beijing 100083, China; shaohuangbian@cau.edu.cn (S.B.); susan@cau.edu.cn (S.S.); sy20243082029@cau.edu.cn (J.Z.); ycxself@cau.edu.cn (C.Y.); 2 College of Science, China Agricultural University, Beijing 100083, China

**Keywords:** tomato leaf disease identification, complex background, multi-scale feature fusion, sample imbalance

## Abstract

In this study, we propose a multi-scale feature fusion network based on an improved RT-DETR model for the efficient detection of tomato leaf disease. Our model combines the multi-scale extended residual module by capturing contextual information at various scales and the multi-scale feature pyramid network by integrating feature information from different levels, which improves feature extraction capability and reduces the interference of complex backgrounds on feature extraction, thereby improving information transmission efficiency and the accuracy of the model. In addition, the novel loss function called adaptive focal loss (AFL) was used, which is based on traditional focal loss with the introduction of attenuation factors to focus the model’s attention to high-loss features to alleviate overfitting and of dynamic weight adjustment mechanisms to focus on the more important features during the training process to improve the overall learning performance. Another significant advantage of AFL is that it can more efficiently improve the detection accuracy on imbalanced datasets than on balanced datasets. These innovations optimized the learning strategy of the model, making AP@0.50 up to 97.9% on detecting the categories of tomato diseases. In addition, this model also achieves the high detection accuracy of 85.4% on other crop diseases. These results provide valuable references for agriculture applications.

## 1. Introduction

Tomato is one of the most widely cultivated horticultural crops worldwide and provides rich nutrients such as vitamin C, lycopene, and various antioxidants, contributing to chronic disease prevention, immune enhancement, and cardiovascular health improvement [1]. However, tomato plants are highly susceptible to a variety of diseases, most of which manifest on the leaves, leading to reduced photosynthetic efficiency, lower yield, and even severe crop loss [2]. Therefore, accurate detection of tomato leaf diseases has become a critical task in smart agriculture [3].

Early research primarily focused on end-to-end Convolutional Neural Networks (CNNs) with feature learning capabilities [4]. Mohanty et al. [5] established a CNN-based PlantVillage benchmark, which significantly advanced the field. Subsequent works integrated CNN architectures into detection frameworks, such as Fuentes et al. [6], who employed dual Faster R-CNN models to detect nine disease and pest categories, and Agarwal et al. [7] proposed a lightweight CNN classifier. Improved faster R-CNN variants further enhanced feature extraction and localization performance [8,9,10].

In recent years, the research focus of tomato leaf disease detection has gradually shifted from solely improving classification accuracy to enhancing robustness, generalization ability, and deployment efficiency in complex field environments [11,12,13,14,15,16]. Badiger et al. [12] integrated optimization algorithms with deep learning to jointly perform leaf segmentation and multi-disease detection, improving the recognition of target regions under complex backgrounds. Lee et al. [16] applied ensemble learning and hyperparameter optimization to further stabilize performance and improve generalization. Similarly, the hybrid ensemble strategies that combine fine-tuned transfer learning models with stacking or traditional machine learning meta-classifiers have been shown to further boost accuracy. For example, Khalid et al. [17] proposed a deep multistacking integrated model that aggregates predictions from several fine-tuned transfer learning networks and employs an XGBoost meta-classifier, achieving high accuracy on multi-crop datasets.

With the increasing influence of Vision Transformers (ViTs) in computer vision, Transformer-based architectures have also been introduced into tomato disease recognition [18,19,20,21,22]. Hossain et al. [18] incorporated multi-scale attention and mobile-friendly designs to enhance field-level recognition performance. Nishankar et al. [23] demonstrated that multi-model fusion and spatio-temporal attention can further improve robustness under natural backgrounds. Some studies have achieved high-precision identification of multiple types of tomato diseases by integrating DenseNet, ResNet, and Transformer structures or improving the training strategy of DeiT [24,25,26].

Moreover, several recent studies have applied a DETR (Detection Transformer) to tomato leaf disease detection for lesion localization and instance segmentation. Wu et al. [27] proposed Spatially Modulated Co-Attention (SMCA) and an enhanced relative position encoding (iRPE) to improve instance segmentation of disease spots on tomato leaves. Wang et al. [28] employed a Swin-DDETR backbone, combined with deformable attention, Meta-ACON activation, and a bidirectional weighted feature pyramid (IBiFPN), achieving real-time detection of small lesions under complex backgrounds. Sun et al. [29] introduced a CSWin Transformer backbone together with a Local Feature Enhance Pyramid (LFEP) and a Multi-Kernel Convolution Module (CMKM), significantly improving detection performance across varied lesion scales and challenging environments.

However, despite substantial progress in tomato leaf disease detection, several challenges remain. First, most existing tomato datasets contain only isolated leaf images with simple or uniform backgrounds, which poorly simulate real-world conditions. Consequently, models trained on such datasets often perform poorly under complex natural environments. Second, class imbalance is prevalent because most samples represent healthy leaves, causing models to bias toward common categories and reducing recognition accuracy for rare diseases. Moreover, although the differences between healthy and diseased leaves are generally significant, subtle variations in color, shape, or texture among different disease types make accurate classification challenging. These limitations hinder the practical deployment of current models in diverse agricultural settings.

To address these issues, this study constructed a new dataset based on the selection and adjustments from two different public tomato datasets (single cut leaf with simple background and multiple leaves in actual environment). In addition, this study proposed a multi-scale dilated residual module and feature pyramid network for feature extraction and fusion based on the RT-DETR model. Finally, the proposed model conducted robustness, generalization, and ablation experiments to evaluate the detection ability. The contributions of this study are as follows.

(1) In view of the challenges of the similarity of diseased leaf features and background complexity, a new dataset was constructed after data augment containing 12,044 images and eight kinds of common tomato leaf diseases to verify the performance of the proposed model. Then, another new dataset containing three imbalance sub-datasets was constructed with the selection from the previously constructed dataset to verify the performance of the proposed model on imbalanced samples.
(2) Finally, the robustness of the model was verified. In addition, this study proposes a new loss function (adaptive focal loss), as well as a random grouping undersampling method to simulate and solve the problem of data imbalance. Experiments show that adaptive focal loss can effectively solve the problem of data imbalance.

## 2. Data Collection and Pre-Processing

The existing open-source tomato leaf disease datasets exhibit deficiencies in both environmental diversity and data quality, which hinder the in-depth development of disease recognition research. One type of dataset is collected in a laboratory setting, featuring a single background, uniform lighting, and consistent perspective. Although it facilitates model validation, it lacks the complexity of real production environments. The other type of dataset is collected in natural scenes, featuring complex backgrounds and diverse leaves. However, it lacks the uniform perspective and clean background provided by laboratory conditions. In addition, many datasets suffer from issues such as varying image quality, numerous noisy samples, and inaccurate annotations, making it difficult for models to generalize across different environments.

**Figure 1:**
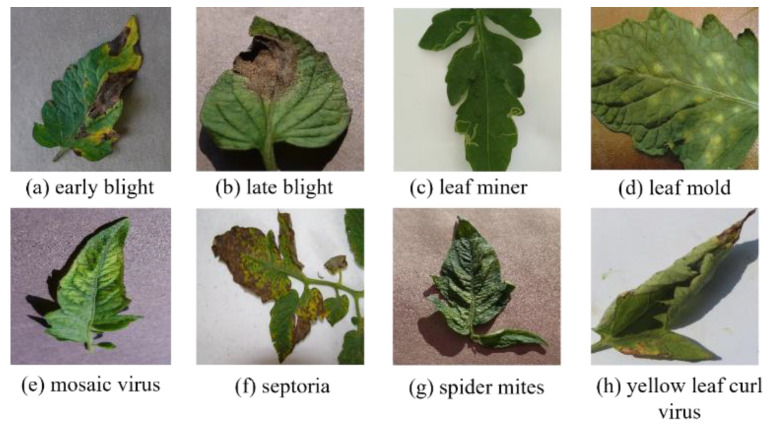
Eight types of disease images in the LAB.

**Figure 2:**
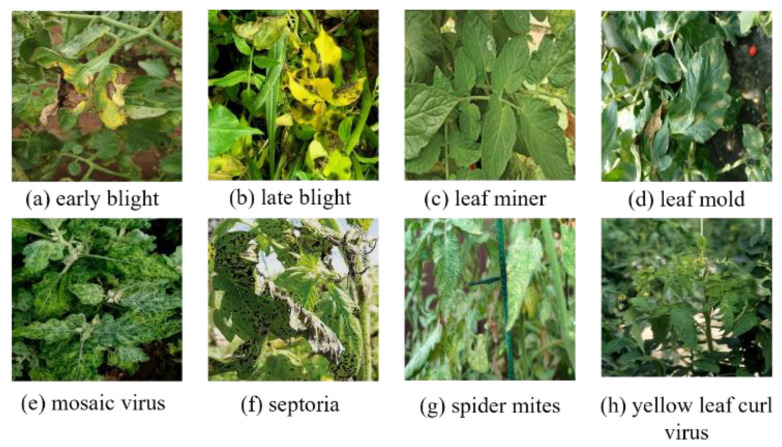
Eight types of disease images in the ENV.

Addressing the aforementioned issues, this study selected samples that met the research requirements from two public datasets [30,31] and constructed two new tomato leaf disease datasets. The dataset includes nine categories (eight types of diseases and one type of health), with a total of 5200 training images. The LAB dataset consists of single leaf images captured in a simple laboratory background with uniform lighting and viewing angles. In contrast, the ENV dataset includes multi-leaf images in different natural scenarios with complex backgrounds, including variable lighting and different perspectives. All images were annotated with bounding boxes using LabelMe v5.6.0 software, and our team supplemented the selection and adjustment process with additional manual validation to ensure accuracy. Figure 1 shows the same eight types of disease images in the LAB. Figure 2 shows the eight types of disease images in the ENV.

**Figure 3:**
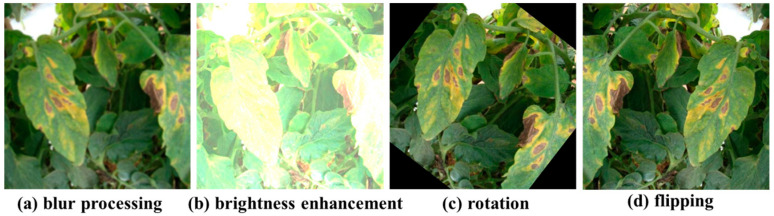
Image enhancement operations.

To generate diverse samples to improve model generalization, we strictly follow the principle of split-first-then-augment: each original tomato dataset (LAB and ENV) with 2600 original images was initially partitioned into independent training and testing sets using unique sample IDs. Each dataset (LAB and ENV) includes 1604 images for testing without augmentation later and 996 images together with their augmentation (6022 images) for training, i.e., only the training images were augmented (including blurring, brightness enhancement, rotation, and horizontal flipping, as shown in Figure 3). This ensures that each dataset contains 7626 images and the segmentation of each dataset is shown in Table 1. In each dataset, the image number of each disease category is shown in Table 2, indicating that the number difference among different categories is not significant. That is to say, the number of samples of different categories in this dataset is approximately balanced.

**Figure 4:**
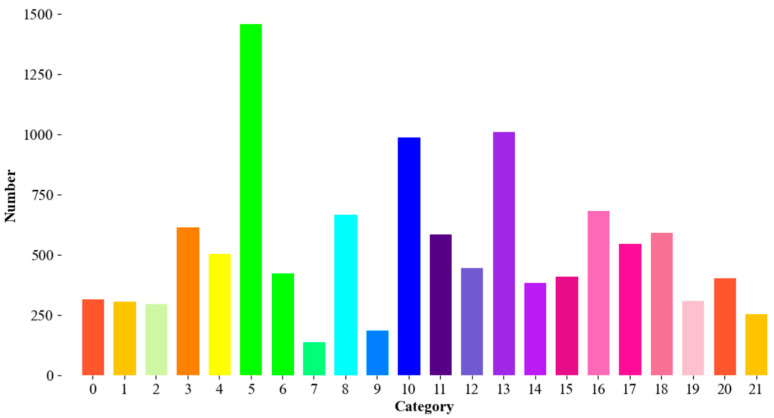
Different categories and the corresponding sample number in the constructed PLANT.

In addition, in this study, in order to verify the generalization of the proposed model, we also constructed another dataset named as PLANT with 13 crops and 22 categories (including tomatoes) through deleting some false samples and performing data enhancement from the original dataset PlantDoc [32]. The constructed PLANT contains 3500 images for training and 150 images for test, and its category distribution is shown in Figure 4.

## 3. Methodology

### 3.1. Overview

**Figure 5:**
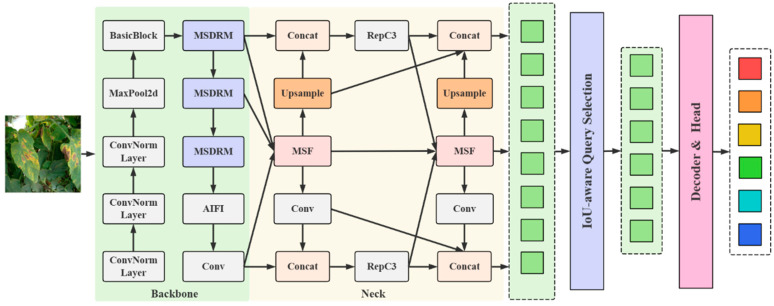
The structure of the proposed MSFF-DETR model.

According to the challenges faced by tomato leaf disease detection, this paper proposes a multi-scale feature fusion DETR (MSFF-DETR) model. The proposed model is optimized based on the RT-DETR-r18 model, and its structure is shown in Figure 5. In the deep layer of the backbone network, a multi-scale dilation residual module (MSDRM) is introduced to replace the original residual block (BasicBlock). The MSDRM includes a dilation-wise residual module (DWR) [33] and a dilated reparam block (DRB) [34]. The DWR module enhances the extraction of multi-scale contextual information and emphasizes simplicity and efficiency at multiple scales. The DRB module is mainly used to enhance the feature extraction capabilities at different levels and alleviate the gradient vanishing problem. The proposed MSDRM converts edge features into local features, which may be ignored in traditional networks, and then fuses them with deep features. This fusion enables the model to dynamically extract information at different spatial levels, enhancing the comprehensiveness of feature extraction. Secondly, this paper proposes a new multi-scale feature pyramid network (MSFPN), which contains a multi-scale focusing module and a context diffusion mechanism. The focusing module contains a parallel deep convolution group to capture multi-scale features. This process makes the features of each scale more detailed. Then, through the context diffusion mechanism, the multi-scale context feature information relationships are diffused to each detection scale. This mechanism ensures information sharing between different scales, making the model more comprehensive and accurate when dealing with multi-classification. In addition, this paper proposes a new loss function, i.e., adaptive focal loss. This loss function dynamically adjusts the IoU threshold so that the model can flexibly adjust its learning strategy for samples of different categories to alleviate the problem of sample imbalance.

### 3.2. Multi-Scale Dilation Residual Module

**Figure 6:**
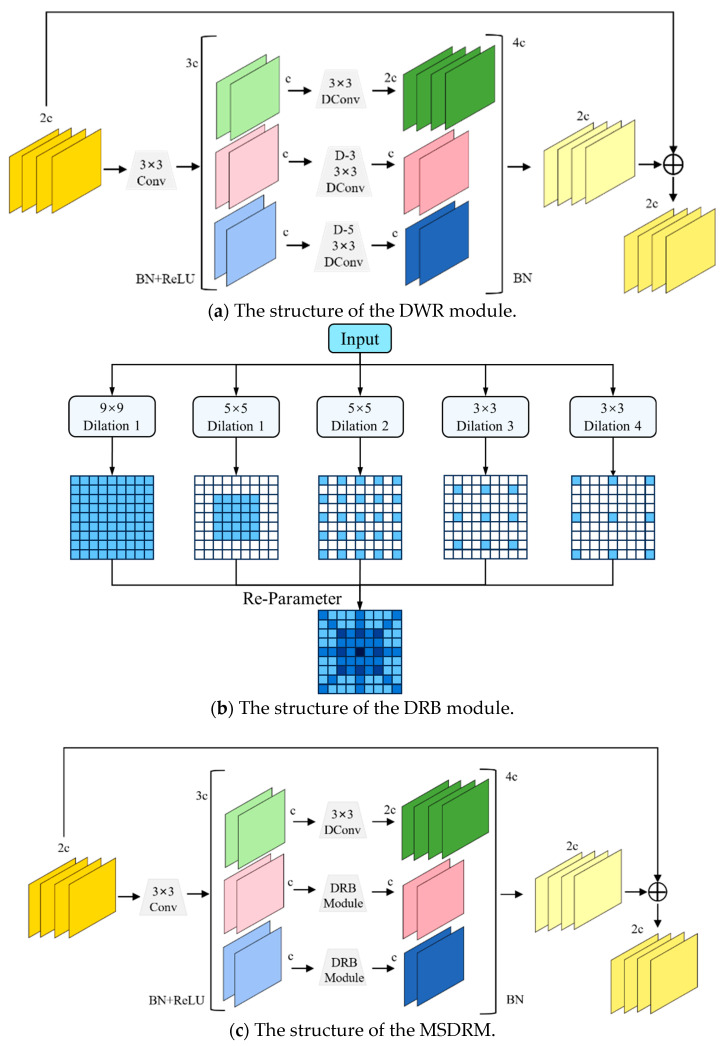
The structure of our proposed MSDRM.

For the problem of multi-scale feature extraction, most methods directly apply deep dilated convolution. However, when the input feature map is complex, it is difficult to effectively extract multi-scale context information due to the unreasonable network structure and hyperparameter settings, so the efficiency of feature extraction is low. In addition, in the process of obtaining multi-scale context information, the fusion of cross-scale features may cause some important local small-scale features to be ignored. In this paper, the DWR module mainly includes two stages: regional residualization and semantic residualization, shown in Figure 6a. The first stage generates regional feature maps of different sizes through simple convolution, and the second stage performs morphological filtering on the regional feature maps to obtain multi-scale features. The DRB module combines large kernel convolution and multiple small kernel convolutions to form a parallel structure, shown in Figure 6b. The large kernel convolution is used to capture global features, while the small kernel convolution helps capture local details. The output of each small kernel convolution is added to the output of the large kernel convolution to integrate features of different scales.

To effectively capture the multi-scale feature information of tomato diseased leaves, we fuse the DWR and DRB modules to propose the MSDRM (shown in Figure 6c), which first performs region residualization to generate regional feature maps of different sizes from the input features. Unlike simple replacement, it selectively reconstructs semantic residualization by retaining the first-path 3 × 3 dilated convolution (dilation rate d = 1) for fine local details while replacing the deeper dilated paths with lightweight DRB sub-modules and each DRB performs efficient reparameterized multi-kernel fusion, followed by pre-fusion morphological filtering (MaxPool − MinPool) on regional features to preserve edge continuity. All the paths are summed after a shared BN and added to the input as residual. The selective DRB substitution (with keeping the output the same as the original DConv module) and filtering fusion strategy effectively expand the receptive fields and especially enhance the edge feature extraction of the leaf disease as well as maintaining computational efficiency by lightweight depth-wise operations.

### 3.3. Multi-Scale Feature Pyramid Network

**Figure 7:**
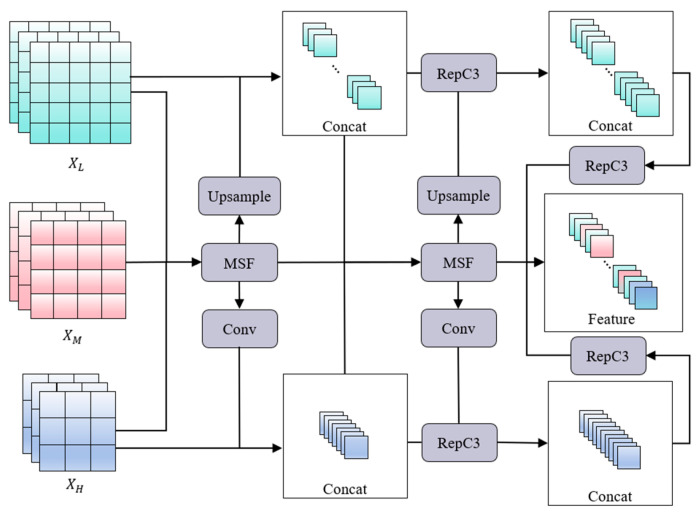
The structure of our proposed MSFPN (multi-scale feature pyramid network).

Traditional feature pyramid structures such as FPN [35], PANet [36], NAS-FPN [37], and BiFPN [38] mainly focus on improving feature flow between adjacent layers through top-down, bottom-up, or weighted bidirectional paths. However, these approaches usually rely on simple summation or weighting strategies, which limit their ability to capture fine-grained features while detecting subtle disease features in agricultural images. For example, the tiny spots and disease characteristics on leaves are often very subtle in complex backgrounds and cross-scale interactions are required. In this study, the proposed MSDRM can meet such requirements by focusing and diffusing the multi-scale features of the different levels. Specifically, we use its output of different layers as the input of the MSFPN and then use the multi-scale focusing (MSF) module, the subsequent context diffusion mechanism (by Upsample and Conv), and another similar operation to fuse the final multi-scale feature. Its structure is shown in Figure 7.

In Figure 7, it is seen that this MSFPN mainly uses two multi-scale focusing modules and a context diffusion mechanism. First, the first cross-scale feature information is focused, and the feature maps of different levels (low-level $X_{L}$, middle-level $X_{M}$, and high-level $X_{H}$) are sent to the MSF module for focusing. Then, the output is upsampled to obtain $X_{UP}$, which is then fused with the low-level features to obtain the cross-scale low-level feature fusion information $X_{R}^{L}$. The formula is as follows:

(1) $$X_{UP}=Up\left(MSF\left(X_{L},X_{M},X_{H}\right)\right)$$

(2) $$X_{R}^{L}=Concat\left(X_{L},X_{UP}\right)$$

Among them, $X_{R}^{L}$ is the first output result of the low-level feature, and R indicates that the result will be used as the input of the RepC3 module. At the same time, the output of the MSF module is subjected to a 3 × 3 convolution operation with a step size of two to obtain $X_{C}$, which is then fused with high-level features to obtain cross-scale high-level feature fusion information $X_{R}^{H}$. The formulas of $X_{C}$ and $X_{R}^{H}$ are as follows:

(3) $$X_{C}=Conv\left(MSF\left(X_{L},X_{M},X_{H}\right)\right)$$

(4) $$X_{R}^{H}=Concat\left(X_{L},X_{C}\right)$$

Among them, $X_{R}^{H}$ is the first output result of the high-level feature, and R indicates that the result will be used as the input of the RepC3 module. Then, the second cross-scale feature information focusing is performed and the output results $X_{R}^{H}$ (the first high-level features) and $X_{R}^{L}$ (low-level features) and the output results of the first MSF module are sent to the second MSF module, and then its output is simultaneously upsampled and convolved to obtain $X_{UP}^{\prime }$ and $X_{C}^{\prime }$. The calculation process is similar to the first cross-scale feature information focusing. Finally, the context diffusion mechanism is used to connect the feature information obtained by the two upsampling operations with the output of the RepC3 module at the lower and higher levels, and the feature information obtained by the two convolution operations relates to the output of the RepC3 module at the higher level. The formulas are as follows:

(5) $$X^{L}=Concat\left(X_{R}^{L},X_{C}^{\prime },X_{C}\right)$$

(6) $$X^{H}=Concat\left(X_{R}^{H},X_{UP}^{\prime },X_{UP}\right)$$

Among them, $X^{L}$ and $X^{H}$ are the final output results of low-level and high-level features. The MSFPN realizes the cross-scale fusion of low-level and high-level feature information through two multi-scale focusing modules and the context diffusion mechanism so that the model can integrate detailed local features with rich global semantic information and enhance the adaptability of the model to complex scenes.

**Figure 8:**
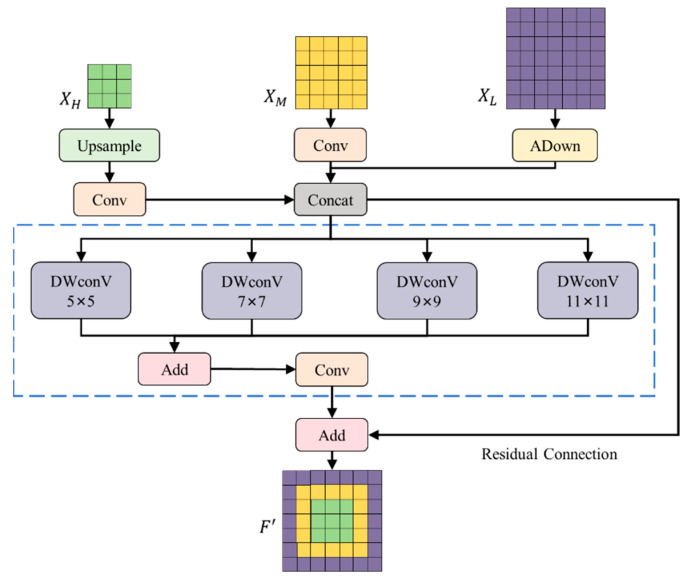
The structure of the MSF (multi-scale focusing) module.

The MSF structure designed in this paper is shown in Figure 8. The input feature maps are $X_{H}$, $X_{M},$ and $X_{L}$. The feature maps are processed and concatenated through upsampling, downsampling, and convolution operations, and then the feature maps are obtained through a set of parallel depth-wise separable convolution (DWconV) and point-wise convolution operations, and finally the final output result is obtained through residual connection.

The specific workflow is as follows. (1) Processing of input data and feature concatenation. The high-level feature $X_{H}$ was upsampled to match the size of the middle and low-level features, and then a 1×1 convolution was used to reduce the number of channels. The 1×1 convolution on the middle-level feature $X_{M}$ and downsampling of the low-level feature $X_{L}$ were performed to extract its representative features. Finally, concatenate the processed high-level, middle-level, and low-level features in the channel dimension to form the following feature map F that integrates multi-scale information.

(7) $$F=Concat\left(Conv\left(Up(X_{H})\right),{Conv}_{1\times 1}\left(X_{M}\right),AD\left(X_{L}\right)\right)$$

(2) Depth-wise classifiable convolution operation. The four depth-wise separable convolutions with different convolution kernel sizes (5×5, 7×7, 9×9, 11×11) were used to extract multi-scale features and process the spliced feature map F. Then, the outputs of the four depth-wise separable convolutions were added element-by-element to obtain the feature map D that integrates multi-scale information. This step can enhance the expressiveness of the feature map and make it contain richer information. The formula of the map D is as follows:

(8) $$D=\sum {DWConv}_{k}\left(F\right)$$

where k∈{5,7,9,11} and ${DWConv}_{k}$ represents the depth-wise separable convolution with a convolution kernel of k×k.

(3) Point-wise convolution and residual connection. The 1×1 point-wise convolution on the fused feature map D was performed to further integrate feature information. Then, the output of the point-wise convolution was added to the original concatenated feature map F element-by-element to form the following output feature map $F^{\prime }$. This residual connection helps with transferring the flow of information, prevents the gradient vanishing problem in deep networks, and improves the performance of the model. The final output contains the residual connection of the original feature map and the processed feature map, thereby retaining the original feature information and enhancing the feature expression ability, providing rich cross-scale information for the further fusion and context diffusion of subsequent features.

(9) $$F^{\prime }=F+{Conv}_{1\times 1}\left(D\right)$$

In contrast, the proposed MSFPN introduces two multi-scale focusing (MSF) modules and a context diffusion mechanism to achieve more effective cross-scale feature integration. Each MSF module performs adaptive fusion of low-, middle-, and high-level features using parallel depth-wise separable convolutions with multiple kernel sizes, allowing the model to aggregate texture and semantic information across receptive fields of different scales. The context diffusion mechanism further strengthens the exchange of information between local and global features, enhancing spatial consistency and contextual perception. Compared with the FPN, our MSFPN emphasizes feature complementarity rather than simple aggregation, and achieves bidirectional multi-scale fusion. These designs enable the model to integrate fine local details with rich semantic context, improving detection performance under complex backgrounds and variable lighting conditions in a real agricultural environment.

### 3.4. Adaptive Focal Loss

In tomato leaf disease datasets, the number of samples for each disease category varies greatly. Rare diseases often have far fewer images than common ones, which causes models to bias toward majority classes during training and neglect minority ones. Existing adaptive focal loss variants have attempted to mitigate sample imbalance and control gradient contributions. For example, focal loss uses a fixed focusing parameter to reduce the impact of easy negatives, but it cannot adapt to changes in sample difficulty over time. Varifocal loss [39] introduces confidence-based weighting; however, its weighting strength remains static throughout training. Slide loss [40] improves adaptivity by employing a sliding threshold, yet it does not explicitly regulate the contribution of high-loss outliers nor incorporate batch-level quality statistics. Consequently, these methods may still overfit noisy or rare-class samples and fail to fully exploit informative medium-quality predictions, which are crucial for imbalanced agricultural datasets.

To solve these limitations, we propose adaptive focal loss (AFL), which enhances varifocal loss with two complementary adaptive mechanisms specifically designed for imbalanced tomato leaf disease datasets. First, by introducing the decay factor $D_{i}$, the model can suppress the focus on high base loss samples and avoid overfitting. The decay factor is as follows:

(10) $$D_{i}=\delta \cdot \left(1-e^{-\frac{i}{\lambda }}\right)$$

where δ and λ are the decay coefficient and the parameter of controlling decay rate (default: 0.999 and 2000, same as those of other loss functions), and i is the number of model updates. Formula (10) indicates that as the update number increases, the decay factor is progressively adjusted from near 0 to approach δ. At early training stages, $D_{i}\approx 0$ allows full gradient updates to explore the loss landscape. As i grows, $D_{i}$ gradually increases, causing all gradients to decay by a factor (≤δ). This design prevents gradient explosion and overfitting, ensuring training stability. Secondly, a dynamic weight adjustment mechanism is used, which includes an auto_iou parameter (batch IoU with lower bound 0.2). This parameter divides the true value ${True}_{i}$ into three intervals with three corresponding weights and dynamically adjusts the weight according to Equation (11).

(11) $${modulating\_weight}_{i}=\left\{\begin{array}{c} 1.0,\;if\;{True}_{i}\leq auto_{iou}-0.1\\ e^{1.0-auto\_iou},\;if\;auto\_iou-0.1{<True}_{i}<auto\_iou\\ e^{1.0-{True}_{i}},\;if\;{True}_{i}\geq auto\_iou \end{array}\right.$$

where $auto_{iou}$ is updated via $auto_{iou}=max\left(D_{i}\cdot {auto}_{iou\_pre}+\left(1-D_{i}\right)\cdot iou\_batch,0.2\right)$, and iou_batch is the average iou of the current batch; ${auto}_{iou\_pre}$ is $auto_{iou}$ calculated for the last time. This mechanism creates a sliding focus band: low-IoU samples receive normal weight (1.0), medium-IoU samples near the threshold are amplified by $e^{1.0-auto\_iou}$ to emphasize boundary learning, and high-IoU samples are down-weighted by $e^{1.0-{True}_{i}}$ to reduce over-emphasis on easy cases. Since ${modulating\_weight}_{i}$ depends only on ground-truth ${True}_{i}$ (not predicted values), it introduces no conflicting gradient signals and cleanly amplifies informative medium-quality predictions—crucial for rare disease detection.

The base loss uses varifocal loss is shown in Equation (12):

(12) $$VFL\left({Pre}_{i},{True}_{i}\right)=-\left[\alpha \cdot \sigma {\left({Pre}_{i}\right)}^{\gamma }\cdot \left(1-{True}_{i}\right)+{True}_{i}\right]\cdot BCE\left({Pre}_{i},{True}_{i}\right)$$

here, α is the scaling factor applied to negative samples (default: 0.75), and γ is the focusing parameter that reduces the weight of easy negatives (default: 2.0).

The final loss formula is shown in Equation (13).

(13) $$Loss=\frac{1}{N}\sum_{i=1}^{N}VFL({Pre}_{i},{True}_{i})\cdot {modulating\_weight}_{i}$$

where BCE is binary cross entropy with logits and ${Pre}_{i}$ is the predicted value. Through ${modulating\_weight}_{i}$, the weight of the sample can be adjusted dynamically to help the model focus on important medium-IoU samples during training and enhance the learning effect on rare classes. The decay factor $D_{i}$ controls the smoothness of the auto_iou threshold but does not scale the final loss. The joint effect is theoretically stable: $D_{i}$ is smooth and monotonic, ensuring the gradual adaptation of the focus band; ${modulating\_weight}_{i}$ is piecewise constant or input-independent in derivative, avoiding instability. Together, they enable balanced and robust optimization of imbalanced tomato leaf disease data.

By jointly leveraging the decay factor and dynamic IoU-based weighting, the proposed AFL achieves time-dependent gradient control and sample-quality-aware weighting, enabling the model to focus on informative medium-IoU samples and enhancing the learning ability for rare disease categories. Unlike previous adaptive losses that mainly rely on fixed thresholds or static weights, the AFL dynamically balances exploration and stability, improving the robustness against noisy labels and biased distributions in imbalanced datasets.

### 3.5. Model Evaluation Indicators

The mAP@0.50 and mAP@0.50:0.95 will be used as the evaluation indicators. The mAP@0.50 refers to the average precision calculated at a threshold of 0.50, which is mainly used to evaluate the detection performance of the model under relatively relaxed conditions. The mAP@0.50:0.95, averaged over the IoU thresholds from 0.50 to 0.95, provides a more rigorous and comprehensive performance evaluation and is stricter than mAP@0.50. In practical applications, mAP@0.50:0.95 can better show the comprehensive ability and robustness of the model. The formulas of the evaluation indicators of mAP are expressed as follows:

(14) $$mAP=\frac{1}{N}\sum_{t=1}^{N}AP\left(t\right)$$

where *N* is the number of thresholds, $AP=\int_{0}^{1}P\left(R\right)dR$, $P\left(R\right)$ is the precision at the summon rate R=TP/(TP+FN)×100% and in which TP shows the number of samples correctly predicted as positive by the model, and FN shows the model’s incorrectly predicted number of samples in the negative class.

## 4. Experiments

### 4.1. Experimental Environment and Model Hyperparameters

The experimental environment used in this study was Windows 11 environment (CPU Intel (R) CORE (TM) i9-10900K, GPU Dual NVIDIA RTX 3090). The framework adopts PyTorch 2.22, the programming language is Python 3.8, and the acceleration calculation adopts CUDA 12.1. Table 3 shows the detailed hyperparameter settings. These settings are based on the original RT-DETR model and have been determined through multiple experiments.

In our study, we explored five different scale network structures of the RT-DETR series [41], namely RT-DETR-R18, R34, R50, L, and X series. These network structures make different tradeoffs between detection accuracy, parameter quantity, and computational complexity to meet the application requirements in different scenarios. Table 4 shows the experimental results of the experimental dataset for different networks of the RT-DETR. RT-DETR-R18 shows the highest accuracy on both LAB and ENV. In addition, RT-DETR-R18 has significant lowest parameters and GFLOPs, which makes it suitable for limited computational resource environments. That is why the RT-DETR-R18 network was chosen as the basic infrastructure in this paper.

### 4.2. Effect of MSDRM on Feature Extraction and MSFPN on Feature Fusion

#### 4.2.1. MSDRM

In the RT-DETR-r18 backbone network, the BasicBlock module is used for downsampling, which may lead to the incomplete acquisition of multi-scale feature information. This study designed an MSDRM (or DRB+DWR) module containing two modules, DRB and DWR, to replace the original BasicBlock module. Table 5 lists the mAP@0.50 and mAP@0.50:0.95 results of different replacement methods on three validation sets. In this study, only adding DRB or DWR can significantly improve the performance of the model. Specifically, the DRB module achieved the mAP@0.50 of 1.1% higher than the original RT-DETR in LAB and 6.9% higher in ENV. Although the improvement in PLANT was relatively small, it was still better than the RT-DETR. The DWR module shows that the mAP@0.50 is 1.3% higher than the RT-DETR in LAB and a similar improvement in ENV. When the DRB and DWR modules are combined to form the MSDRM, the performance is further improved. The mAP@0.50 of the MSDRM shows an increase of 2.1% for LAB, 10.7% for ENV, and 8.1% for PLANT compared to the RT-DETR. These results show that the MSDRM effectively combines the advantages of DRB and DWR to improve the detection accuracy. In addition, the parameters and GFLOPs of the MSDRM show that it does not significantly increase the model complexity compared to the RT-DETR.

#### 4.2.2. MSFPN Module

**Figure 9:**
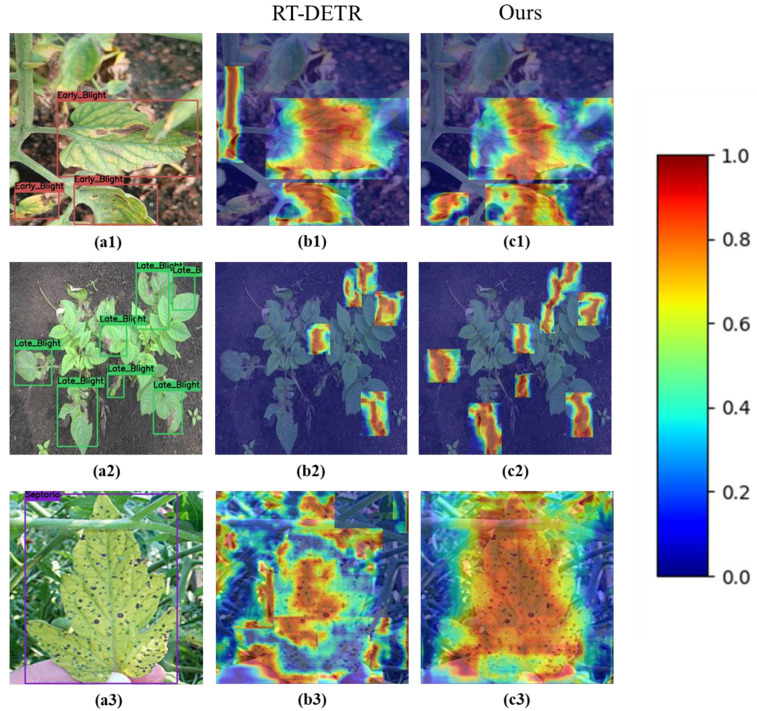
Heat map display of different models on ENV dataset: (**a1**–**a3**) for the labeled real diseased leaves, (**b1**–**b3**) and (**c1**–**c3**) for the corresponding heatmaps by RT-DETR model and our model, respectively.

In this study, the MSFPN structure is used in the neck part of the RT-DETR to focus and diffuse the multi-scale information of different levels obtained by the MSDRM and other convolution operations to achieve multi-scale feature information fusion. To show the effect of the improved model more intuitively, a heat map generated by Grad-CAM++ was used to highlight the areas that the model focuses on in the image, as shown in Figure 9. The red in the heat map indicates the high probability area where the model believes the diseased objects exist. It is seen that our model pays attention to the diseased leaf area more carefully and obtains more accurate feature information. For example, Figure 9(b1,c1) shows that the RT-DETR model misidentified the stem as diseased leaves and did not identify the diseased leaves in the left corner of the image, while our model can effectively remedy the two aforementioned deficiencies. Figure 9(b2,c2) shows that several diseased leaves were not identified by the RT-DETR, but our model identified them accurately. Figure 9(a3–c3) shows that our model can more accurately distinguish diseased leaves from complex backgrounds. These results indicate that the MSFPN structure pays attention to key features of diseased leaves, enhances the response to different disease points, and selectively fuses this information. This design enables the model to better capture the details of similar leaf disease features and reduces the interference of background features to improve detection accuracy.

### 4.3. Effect of Afl Modules

In this paper, the adaptive focal loss (AFL) function is proposed, which adjusts the weights based on the similarity (such as IoU) between the predicted results and the true labels. It introduces an attenuation factor and a dynamic weight adjustment mechanism to suppress excessive attention to high initial loss samples while enhancing attention to important samples during training. It can properly improve the detection performance on datasets, especially on the imbalanced categories.

The sample imbalance, i.e., the number of samples in different categories varies greatly, is a common issue for diseased tomato categories. In this study, the proposed AFL function can be used to solve this problem to some extent. For example, for the minority disease category, the loss weights are amplified, making the model pay more attention to the learning of these samples. For the majority category disease samples, the loss weights are suppressed to balance the learning of different category samples. To verify the effectiveness of AFL on imbalanced samples, we proposed an improved random sampling technique to obtain the imbalanced dataset based on the previously constructed datasets of LAB and ENV (here they are regarded as balanced datasets because the maximum sample ratio among the categories is only about 2:1). Specifically, we designed a random grouping method based on the partial sampling strategy [42] and random grouping [43]. Then, from the two datasets of LAB and ENV, the nine disease categories were randomly divided into three groups and different random proportions were assigned to each group to obtain the imbalanced datasets. Table 6 shows the constructed imbalanced datasets (Exp-1, Exp-2, and Exp-3) with the random category grouping and sample proportion distribution. The categories of the first group are No. 3, 5, and 7 with the corresponding sample proportions of 0.1, 0.2, and 0.3 in their own categories (i.e., the sample quantity is 136, 391, and 324). A similar distribution was applied to the second and third groups.

**Figure 10:**
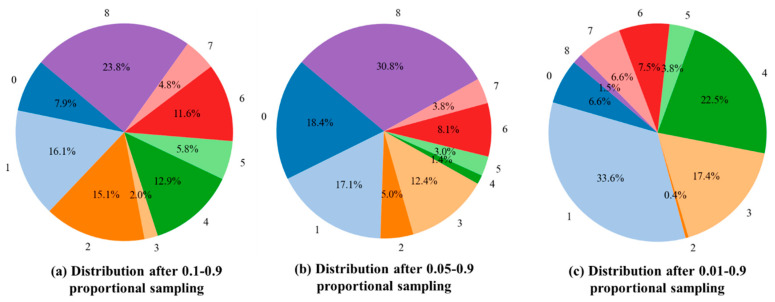
Comparison of category distribution under different proportions of (**a**) 0.1–0.9, (**b**) 0.05–0.9, and (**c**) 0.01–0.9.

Then, pie charts were conducted to visualize the sample proportions of each category shown in Figure 10. The pie chart clearly shows the imbalanced distribution of each category. For example, in Figure 10a–c, the ratio of the highest and lowest proportion is 11.9: 1 (23.8%: 2.0%), 22: 1 (30.8%: 1.4%), and 84: 1(33.6%: 0.4%), which means that the imbalance degree of the above three datasets becomes stronger from Exp-1 to Exp-3. In addition, the proportion of the same category varies across different groups. For example, the proportion of the category “8” in Figure 10a,b is significantly larger than in Figure 10c. The category “4” and class “0” have a similar imbalanced distribution. Table 7 shows the division of the constructed imbalanced dataset.

**Figure 11:**
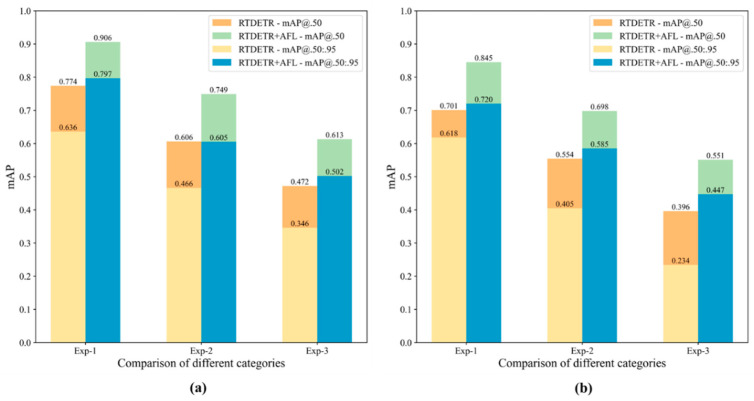
The test results of RT-DETR and RT-DETR+AFL on the imbalanced datasets on (**a**) LAB_Imb and (**b**) ENV_Imb.

Figure 11 shows the experimental results of the RT-DETR and RT-DETR+AFL on the imbalanced datasets of LAB and ENV. Through the combination and comparison of Figure 11a,b, it shows that the RT-DETR+AFL model shows better detection ability and robustness than RT-DETR on these imbalanced datasets, and especially that the AFL module conducts better performance at the lower imbalance degree of categories (Exp-1). As the imbalanced degree of the datasets (i.e., Exp-2 and Exp-3) increases, the detection performance of both the RT-DETR and RT-DETR+AFL gradually decrease (for example, for the Exp_1 in LAB_Imb, the mAP@0.50 of RT-DETR+AFL decreases to 0.613 from 0.906). The comparison of Figure 11a,b shows that both the RT-DETR and RT-DETR+AFL conduct more accuracy detection on LAB_Imb than on ENV_Imb, which is mainly due to the complex backgrounds in the ENV_Imb, reducing the detection accuracy.

### 4.4. Noise Experiment

**Figure 12:**
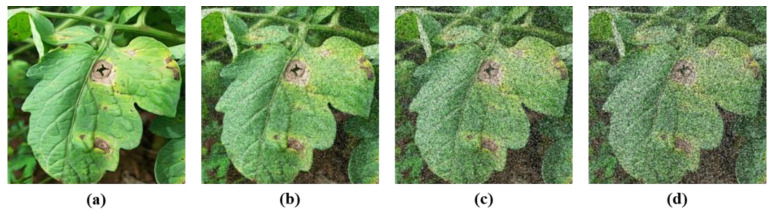
Different proportions of noise of (**a**) 0%, (**b**) 5%, (**c**) 15%, (**d**) 25%.

To evaluate the ability of the proposed model to resist unknown noise (i.e., the noises not included in augmented training datasets), varying proportions of salt noises were injected into the test images in LAB, which features relatively simple backgrounds to facilitate controlled comparison. Specifically, 600 images in LAB are used as test samples and injected with 0 to 25% (2.5% increase each time) of salt noise into each image. Figure 12 shows the images after adding 0, 5%, 15%, and 25% salt noise, indicating that the injection of different proportions of noise make the disease features more difficult to distinguish.

**Figure 13:**
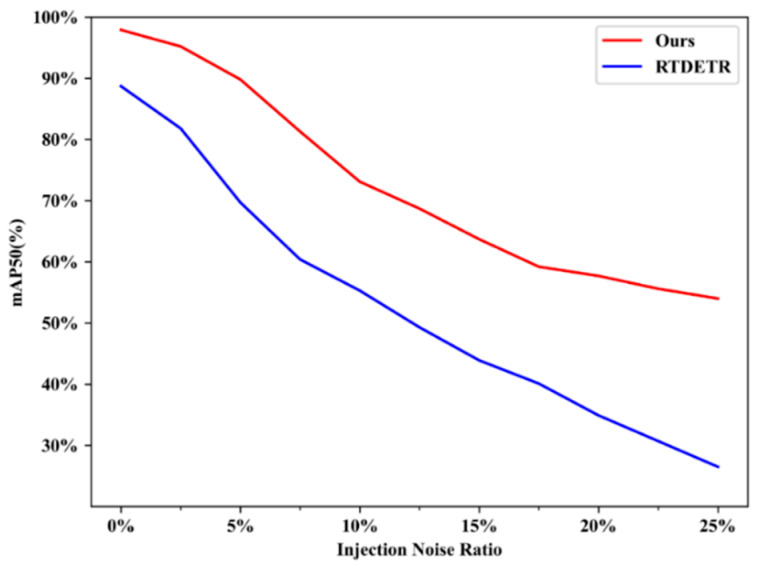
Comparison of our model and RT-DETR on LAB with different proportional noise.

Figure 13 shows the mAP@0.50 comparison of our model and the RT-DETR on LAB on the same test set after adding noise. The results indicate that the detection accuracy of our model decreases more slowly than that of the RT-DETR, especially when the injected noise exceeds 10%, showing that our model has the stronger ability to resist unknown noises.

### 4.5. Comparison Experiment

This study has made a series of improvements to the original RT-DETR model, aiming to improve the accuracy and efficiency of tomato leaf disease detection. In general, YOLO series are commonly used in anchor-based detection frameworks because of their fast detection speed and excellent performance, such as YOLOv8-m, YOLOX-m [44], YOLOv9-m [45], YOLOv10-b [46], YOLOv11-l [47], and YOLOv12-m [48]. The comparison of our model with anchor-based models (including RetinaNet [49], Faster R-CNN [50]) and typical anchor-free DETR series models (including RT-DETR series, DINO-DETR [51], Sparse DETR [52], Deformable DETR [53]) were conducted to validate the performance of our model.

In this paper, each of the three constructed datasets have their own data characteristics, showing different application scenarios and detection challenges. The first dataset (LAB) is an experimental environment dataset mainly containing leaf images without complex backgrounds, suitable for evaluating the detection performance of the model under ideal conditions. The second dataset (ENV) is a real agricultural environment dataset containing complex backgrounds and different lighting conditions, used to evaluate the robustness of the model in practical applications. The third test set (PLANT) is a dataset of various plant diseases, including not only tomatoes but also disease images of other plants, used to evaluate the model’s generalization ability and multi-class detection performance.

Table 8 shows the comparative results of different models on these three datasets (negligible standard deviation). Among the YOLO series, YOLOv8-m on the LAB shows its highest detection accuracy under ideal conditions. However, there is still improvement in YOLO series models when dealing with complex backgrounds (ENV) or multi-plant diseases (PLANT). RetinaNet performs better than most of the YOLO series on the ENV. However, RetinaNet has a higher parameter count (36.496 M) and computational complexity (130 GFLOPs) than the YOLO series, which limits its application in resource-constrained environments.

In anchor-free models, VarifocalNet and RT-DETRv2 have poor performance on all the three datasets. The RT-DETR-r18 and RT-DETRv3 perform well on LAB and ENV, but poorly on the PLANT, indicating their poor generalization. The DINO-DETR shows better detection capability in the three datasets, but with the most parameters and the highest computation complexity (GFLOPs). The Sparse DETR and Deformable DETR (latest models) show an overall better performance than the DINO-DETR with higher accuracy and lower parameters and computation complexity. In contrast, our model shows the best overall performance on all the three datasets, indicating that our model more efficiently detects leaf diseases in real agriculture scenarios with better generalization and lower computation complexity, which can be effectively extended to other plants and easily embedded to edge devices.

### 4.6. Ablation Experiments

**Figure 14:**
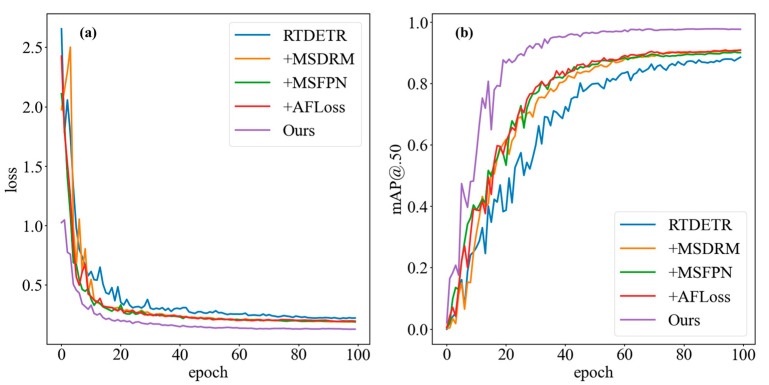
The comparison of the loss (**a**) and mAP@0.50 (**b**) curves of RT-DETR and with different modules on LAB.

In order to illustrate the impact of different modules on the model, we conducted the ablation experiments under the same training environment and hyperparameters. We used the RT-DETR model as the baseline and added different improved modules on this basis. Figure 14 shows the ablation experiment comparison of the RT-DETR model and its different added modules. Figure 14a shows that the RT-DETR loss fluctuates greatly, especially in the 0–50 epochs stage, indicating that the model is not adaptable to the data in the early stage. After the introduction of different modules, although the loss still fluctuates during the 0–20 epochs period, its amplitude is significantly smaller than that of the RT-DETR, which shows that the new modules improve the stability of the model and enable it to adapt to the data faster. Starting from 40 epochs, the loss gradually stabilizes, indicating that the model has converged well and that the parameter update tends to be stable. Our model shows higher stability in terms of loss, with the smallest fluctuation amplitude, showing better stability and convergence. Figure 14b shows that the curve of the RT-DETR, with fluctuations, has not even reached the best performance at 100 epochs. However, our model has stabilized after 60 epochs, showing that our model is able to utilize data more effectively, thereby achieving higher average precision. These ablation experiments clearly show that our model has better stability and convergence in loss and mAP@0.50. This stability and convergence are crucial for model deployment and performance prediction in practical applications to reduce the fluctuation of the model under different datasets or environmental changes and improve the reliability and robustness of the model.

Table 9 shows the ablation experiment comparison with adding different modules on different datasets. It shows that the mAP@0.50 is significantly improved on the three datasets with adding the MSDRM on the RT-DETR, which is because the MSDRM can effectively extract multi-scale features, enabling the model to better capture the detailed information of the object and thereby improving the detection performance. After further introducing the MSFPN, the detection accuracy is further improved, because the MSFPN module can enhance the model’s understanding of object diversity by fusing and focusing on multi-scale feature information, thereby improving the accuracy and robustness of detection. In addition, with continuously adding the AFL module, the mAP@0.50 is further improved to a higher accuracy (0.917), indicating its effective contribution. When the above three modules are combined at the same time (i.e., our model), the detection accuracy is the highest compared with those of only adding a single module or two modules together, although the parameters and GFLOPs of our model are slightly increased. This result fully proves that our model has the highest detection ability on different leaf diseases, which has application prospects in real agriculture scenarios.

Among the ablation experiments, due to its prominent characteristics, the AFL module is dynamically adjusting the attention weight on the samples of imbalanced categories, which is already shown in Section 4.3 (*Effect of AFL modules*). The comparison of AFL on the balanced and imbalanced datasets is shown in Table 10. The more significant increase in the accuracy after adding the AFL module on LAB_Imb and ENV_Imb (13.2% and 14.4%) than on LAB and ENV (3% and 11.8%) shows the better performance of the AFL module on the imbalanced datasets, indicating that AFL can significantly alleviate the negative impact of data imbalance on model performance. By dynamically adjusting sample weights, AFL helps the model pay more attention to the samples of minority categories during training. Even in ENV_Imb with complex backgrounds, the AFL module can help the model to obtain a high detection accuracy. It should be pointed out that when the sample imbalanced ratio is too large (for example, large than 20:1), AFL will not be able to save the model to a high level of accuracy.

**Figure 15:**
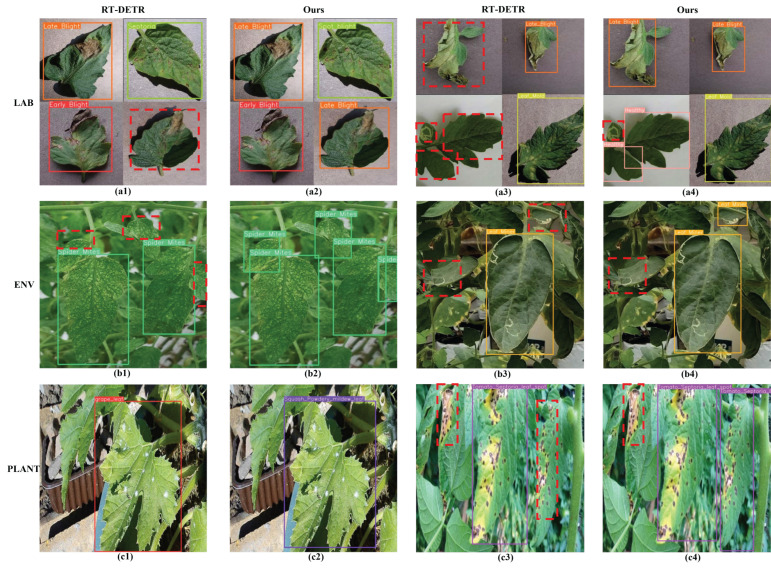
Visualization of detection results for RT-DETR and our model on different datasets: (**a1**–**a4**) for LAB, (**b1**–**b4**) for ENV, (**c1**–**c4**) for PLANT.

Figure 15 shows the visualization results of the baseline RT-DETR model and our model on the three datasets, in which the missed detection areas (including healthy and diseased) are marked in dashed red lines. It is clear that in the LAB dataset, our model only missed one disease (leaf mold in Figure 15(a4), while the RT-DETR failed to detect multiple diseases (early blight in the lower right corner of Figure 15(a1), and leaf mold and healthy in the lower left corner of Figure 15(a3)). Furthermore, on the ENV dataset, the RT-DETR missed more diseased leaves than our model. On the PLANT dataset, our model can simultaneously detect crop names and diseases without false detection (through confirming them with the original labels), with only one missed detection (the red bold part in Figure 15(c4)), indicating the significantly higher detection accuracy of our model than the RT-DETR. Therefore, our model exhibits superior detection performance across different datasets.

### 4.7. Deployment Feasibility and Runtime Analysis

To evaluate the real-time capability and deployment feasibility of our model for smart agriculture applications, runtime benchmarks were conducted on an NVIDIA RTX 3090 GPU (batch size = 1, input size = 640 × 640). Table 11 summarizes the runtime performance comparison of the three datasets. It is seen that the baseline RT-DETR-r18 has an inference latency of about 15 ms per image (or 65 FPS), GPU memory usage peak of 0.24 GB, and a size of model files of 38.6 MB. The corresponding values of our model are 19.8 ms per image (i.e., 50 FPS), 0.52 GB, and 45.6 MB, respectively. It shows that compared with the baseline model, our model keeps good real-time performance, suitable for lightweight deployment on some embedded devices.

## 5. Conclusions

In this paper, the three datasets, LAB (simple backgrounds), ENV (complex natural settings), and PLANT (cross-crop diseases), were constructed to systematically study the proposed model’s adaptability across varying conditions. The proposed MSFF-DETR model (based on the RT-DETR) integrates a multi-scale dilated residual module and a multi-scale feature pyramid network to improve multi-scale feature extraction and information flow. Coupled with an adaptive focus loss function tailored for class imbalance, our model achieves higher accuracy, generalization, and efficiency than the comparative models, showing strong potential for practical agricultural applications.

Despite the advantages, this model still has several limitations and needs further improvement: (1) its computation complexity to be decreased for real application device deployment; (2) its extended application on other crops and real complex agriculture background to be improved. In order to overcome these limitations, this model can be further optimized by using a lightweight network to improve its efficiency for edge deployment and by adding more data of multiple crops to avoid overfitting and improve its generalization.

## Figures and Tables

**Table 1:** The dataset segmentation.

Dataset	Name	Ratio	Quantity
LAB	Training set	0.8	6022
	Test set	0.2	1604
ENV	Training set	0.8	6022
	Test set	0.2	1604

**Table 2:** Sample class name, category number, and quantity.

		LAB	ENV
Category	Name	Quantity	Quantity
0	Early Blight	998	922
1	Healthy	1000	1001
2	Late Blight	1201	1325
3	Leaf Miner	900	972
4	Leaf Mold	1200	1202
5	Mosaic Virus	1100	1338
6	Septoria	1100	1229
7	Spider Mites	700	849
8	Yellow Leaf Curl Virus	1500	1552

**Table 3:** Training hyperparameters.

Parameter	Value
Image Size	640 × 640
Initial LR	0.01
Weight Decay	0.0005
Warm-up	Linear from 0 to 0.01 over first 3 epochs
Final LR Factor	lrf = 0.1
Final LR	0.001
Optimizer	SGD
Data Augmentation	HSV (*p* = 0.7, h = 0.015, s = 0.4, v = 0.3), Horizontal Flip (*p* = 0.5)
Batch Size	4
Epoch	100

**Table 4:** Experimental results of different scale networks of RT-DETR series.

	mAP@0.50	mAP@0.50:0.95	mAP@0.50	mAP@0.50:0.95	Parameters (M)	GFLOPs
RT-DETR Series	LAB		ENV			
R18	0.887	0.784	0.801	0.749	19.838	54.7
R34	0.870	0.749	0.789	0.723	31.118	88.8
R50	0.885	0.762	0.791	0.734	41.972	129.6
L	0.873	0.741	0.787	0.725	32.002	103.5
X	0.875	0.745	0.800	0.747	65.486	222.5

**Table 5:** Different replacement methods for the MSDRM.

	LAB		ENV		PLANT			
Name	mAP @0.50	mAP @0.50:0.95	mAP @0.50	mAP @0.50:0.95	mAP @0.50	mAP @0.50:0.95	Parameters (M)	GFLOPs
RT-DETR	0.887	0.784	0.801	0.749	0.622	0.436	20.093	58.3
+DRB	0.898	0.805	0.870	0.810	0.638	0.441	15.491	54.7
+DWR	0.900	0.811	0.870	0.813	0.631	0.434	24.771	62.1
+DRB+DWR	0.908	0.832	0.908	0.843	0.703	0.559	21.343	59.4

**Table 6:** Imbalanced datasets from the random grouping of the categories with different sample distribution proportions.

		1st Group	2nd Group	3rd Group
Exp-1	Category	[3, 5, 7]	[0, 4, 6]	[1, 8, 2]
	proportion	[0.1, 0.2, 0.3]	[0.4, 0.5, 0.6]	[0.7, 0.8, 0.9]
	quantity	[136, 391, 324]	[534, 875, 787]	[1092, 1610, 1021]
Exp-2	Category	[4, 5, 2]	[7, 6, 1]	[3, 8, 0]
	proportion	[0.05, 0.1, 0.15]	[0.2, 0.3, 0.4]	[0.5, 0.6, 0.7]
	quantity	[60, 132, 220]	[170, 358, 762]	[553, 1370, 819]
Exp-3	Category	[5, 8, 2]	[0, 6, 7]	[3, 4, 1]
	proportion	[0.01, 0.05, 0.1]	[0.2, 0.3, 0.4]	[0.7, 0.8, 0.9]
	quantity	[164, 66, 19]	[285, 323, 284]	[746, 966, 1446]

**Table 7:** The sample distribution of the imbalanced datasets.

Name	Number	LAB_Imb		ENV_Imb	
		Training Set	Test Set	Training Set	Test Set
Exp-1	6770	3034	293	3056	387
Exp-2	4444	1998	208	2006	232
Exp-3	4299	1929	230	1940	200

**Table 8:** Results of the comparison experiments. The dashed lines separate Anchor-based and Anchor-free models.

		LAB		ENV		PLANT			
	Name	mAP@0.50	mAP@0.50:0.95	mAP@0.50	mAP@ 0.50:0.95	mAP@0.50	mAP@ 0.50:0.95	Parameters (M)	GFLOPs
Anchor-based	Yolov8-m	0.821	0.711	0.882	0.839	0.565	0.425	25.861	79.1
	Yolov9-m	0.802	0.736	0.752	0.692	0.483	0.363	20.174	77.6
	Yolov10-b	0.766	0.705	0.757	0.714	0.503	0.387	20.484	98.9
	Yolov11-l	0.767	0.709	0.790	0.742	0.459	0.345	25.327	87.4
	Yolov12-m	0.789	0.726	0.760	0.704	0.539	0.399	19.626	60.2
	YoloX-m	0.866	0.736	0.767	0.692	0.345	0.206	25.331	73.8
	RetinaNet	0.838	0.727	0.859	0.802	0.697	0.546	36.496	130
	Faster R-CNN	0.918	0.786	0.899	0.856	0.756	0.650	41.289	134
Anchor-free	RT-DETR-r18	0.887	0.784	0.801	0.749	0.622	0.436	20.093	58.3
	RT-DETRv2	0.782	0.729	0.723	0.696	0.552	0.434	20.111	61.2
	RT-DETRv3	0.827	0.700	0.807	0.705	0.404	0.280	21.160	63.7
	VarifocalNet	0.751	0.654	0.680	0.590	0.621	0.457	32.231	121
	DINO-DETR	0.827	0.738	0.914	0.815	0.724	0.541	47.550	179
	Sparse DETR	0.900	0.892	0.886	0.851	0.783	0.659	41.182	139
	Deformable DETR	0.890	0.881	0.843	0.802	0.828	0.701	40.312	137
	MSFF-DETR	0.979	0.928	0.970	0.910	0.854	0.706	22.316	67.1

**Table 9:** Test results of the ablation experiments.

	Modules			mAP@0.50				
	MSDRM	MSFPN	AFL	LAB	ENV	PLANT	Parameter (M)	GFLOPs
RT-DETR				0.887	0.801	0.622	19.883	57.0
Exp	√			0.908	0.908	0.703	21.058	57.9
Exp		√		0.907	0.910	0.737	22.246	66.1
Exp			√	0.917	0.919	0.759	19.883	57.0
Exp	√	√		0.952	0.945	0.801	22.316	59.6
Exp	√	√	√	0.979	0.970	0.854	22.316	59.6

**Table 10:** The performance comparison of AFL module on the balanced and imbalanced datasets.

Name	Balanced Datasets		Imbalanced Datasets	
	LAB	ENV	LAB_Imb	ENV_Imb
RTDETR	0.887	0.801	0.774	0.701
RTDETR + AFL	0.917	0.919	0.906	0.845
Increase	3%	11.8%	13.2%	14.4%

**Table 11:** Runtime performance comparison of RT-DETR-r18 and our model on the three datasets.

Dataset	Model	Size (MB)	GPU Mem. (GB)	Latency (ms/img)	FPS
LAB	baseline	38.6	0.243	15.26	65.5
	ours	45.6	0.519	19.80	50.5
ENV	baseline	38.6	0.243	15.54	64.3
	ours	45.6	0.519	19.97	50.1
PLANT	baseline	38.6	0.243	15.17	65.9
	ours	45.6	0.519	19.57	51.1

## Data Availability

The original contributions presented in this study are included in the article. Further inquiries can be directed to the corresponding author.
